# Daily diurnal temperature range associated with outpatient visits of acute lower respiratory infection in children: A time-series study in Guangzhou, China

**DOI:** 10.3389/fpubh.2022.951590

**Published:** 2022-10-20

**Authors:** Zhigang Zhang, Debo Xu, Jiamin Chen, Qiong Meng, Zhenyu Liang, Xiao Zhang

**Affiliations:** Department of Pediatrics, Guangdong Second Provincial General Hospital, Guangzhou, China

**Keywords:** diurnal temperature range, acute lower respiratory infection, China, children, time series analysis

## Abstract

**Background:**

Diurnal temperature range (DTR) has been increasingly recognized as a risk factor for mortality and morbidity, but the association between DTR and acute lower respiratory infection (ALRI) outpatient visits has not been examined among children in China.

**Methods:**

A total of 79,416 ALRI outpatient visits among children were obtained from the Guangdong Second Provincial General Hospital between 2013 and 2019. DTR was calculated by taking the difference between the maximum and the minimum temperatures. Generalized additive models using a quasi-Poisson distribution were used to model the relationship between DTR and ALRI outpatient visits.

**Results:**

Diurnal temperature range was significantly associated with elevated risks of ALRI outpatient visits: the excess risks (ERs) and 95% confidence intervals (CIs) were 2.31% (1.26, 3.36%) for ALRI, 3.19% (1.86, 4.54%) for pneumonia, and 1.79% (0.59, 3.01%) for bronchiolitis, respectively. Subgroup analyses suggested that the associations were significantly stronger during rainy seasons (ER for ALRI: 3.02%, 95% CI: 1.43, 4.64%) than those in dry seasons (ER for ALRI: 2.21%, 95% CI: 0.65, 3.81%), while no significant effect modifications were found in sex and age groups.

**Conclusion:**

Diurnal temperature range may elevate the risk of ALRI outpatient visits among children in China, especially during rainy seasons. Public health policies are needed to mitigate the adverse health impacts of DTR on children.

## Introduction

Lower respiratory tract infection primarily includes pneumonia and bronchiolitis, and it is the leading cause of under-five mortality across the globe (1, 2). A comprehensive analysis of respiratory disease burden in China estimated that 55.8 million lower respirations occurred in China in 2019, representing a 7.7% increase from the number in 2010 (3–5). Considering the mortality and disease burden of the lower respiratory infection among children, it is important to understand and quantify its risk factors and subsequently inform targeted policy making (6, 7).

Climate change has been recognized as the single biggest threat to human health (8, 9). The detrimental health impacts of climate change are increasingly concerning due to rapid global warming and the recent high prevalence of extreme climate events (10–12). Several empirical studies reported the loss of mortality and morbidity of respiratory diseases attributed to heat stress and heat strokes (13). On the other hand, cold spells were reported to be significantly associated with elevated all-cause mortality but not with respiratory diseases (odds ratio 1.21, 95% confidence interval [CI]: 0.97 to 1.51) (14). However, there is little research on the association of diurnal temperature range (DTR), a major index denoting temperature variability (15), with lower respiratory disease outpatient visits in middle-income countries including China.

In this time-series analysis of acute lower respiratory infections outpatient visits in a large tertiary hospital in Guangzhou, China, we propose to quantify the association between DTR and the outpatient visits of ALRI. We hypothesize that DTR can elevate the incidence of outpatient visits with ALRI.

## Methods

### Study area

Guangzhou, in Southern China, belongs to a subtropical humid-monsoon climate, and the annual mean temperature is 22°C and average rainfall is 1,500–2,000 mm, with 18.6 million people in 2020. Guangzhou city has a maritime monsoon climate with an average annual temperature of 22°C, and an average relative humidity of 80%. As the capital city of Guangdong Province, the large number of residents yielded sufficient statistical power and long panels of observation, and the health outcome data have higher quality (16).

### Acute lower respiratory infection data

The data about ALRI outpatient visits from February 2013 to December 2019 were obtained from the Guangdong Second Provincial General Hospital, which is one of the largest tertiary hospitals in Guangzhou, China (17). This administrative database set up by the hospital regularly accumulates data on outpatient visits in the hospital, including demographics, date of visit, and diagnosis. The ALRI outpatient visits were defined based on the International Classification of Diseases, Tenth Revision (ICD-10): ALRI (J12–J18 and J20–J22), pneumonia (J12–J18), and bronchiolitis (J20–J21) (3, 4, 18, 19).

### Meteorological and air pollution data

Meteorological data in this study were collected from the National Weather Data Sharing System (http://data.cma.cn/), including daily maximum, mean, and minimum temperature, and relative humidity. Daily meteorological data across the Guangzhou station were used to reflect the general population's daily exposure. The meteorological data were collected from national regular monitoring stations and there were no missing data. Following the definitions of several previous studies (15, 20, 21), DTR was calculated by taking the difference between the maximum and the minimum temperatures on the same day.

Considering the potential confounding effects of air pollution on the relationship between DTR and ALRI outpatient visits, we obtained daily average concentrations of air pollution, including fine particulate (PM_2.5_), PM_10_, nitrogen dioxide (NO_2_), and sulfur dioxide (SO_2_) in Guangzhou during the study period (22–24). The average of 11 fixed air monitoring stations in Guangzhou was used as the daily mean concentrations for each air pollutant. The fluorescence, chemiluminescence, and ultraviolet were used to measure NO_2_ and SO_2_; and the beta ray attenuation method was used to measure PM_2.5_ and PM_10_ (25).

### Statistical models

Following the study design of a prior time-series study (26), the association between DTR and ALRI was estimated using generalized additive models (GAM) with quasi-Poisson distribution (27). Public holidays (PHs) and days of the week (DOW) were adjusted for as dummy variables in the models. Temporal trends, daily mean temperature, and relative humidity were controlled for as natural cubic splines (28). Degree of freedom (df) was selected by referring to Akaike Information Criterion (AIC), and we chose 5 df per year for temporal trends and 3 df for the daily mean temperature and relative humidity. The model was defined as below:


(1) $$\begin{array}{l} \log\left[E\left(Y_{t}\right)\right]=\beta^{*}DTR+s\left(t,df=6/year\right)\\ +s\left(Temp_{03},df=6\right)+as.factor\left(DOW\right)\\ +s\left(RH,df=3\right)+as.factor\left(PH\right) \end{array}$$

where E(Y_t_) is the expected daily number of ALRI on day t, β means the coefficient of the DTR, s() represents a smoothing function, t is the adjustment for long-term and seasonal trends, Temp_03_ is the moving average for the previous 4 days' temperature, DOW is an indicator for the day of the week, RH presents the humidity on the current day, and PH represents a binary variable for the public holiday.

Due to the potentially delayed adverse impacts of DTR, different lag structures were assessed to examine any potential lag effects. In the single-lag day models, we begin with the same day (lag0) up to seven days lag (lag7). In multi-day lags models, we considered the accumulated effects (moving averages for the current day and the previous 1–5 days [lag01, lag02, lag03, lag04, and lag05]).

### Stratified analyses

To examine whether the adverse impacts of DTR on ALRI differed by sex (male vs. female), age group (age <5 vs. age 5–14), and season (dry vs. rainy), we conducted subgroup analyses stratified by these factors. Due to the tropical monsoon climate, the dry season was defined as the period from November to April of the following year, and the rainy season was from May to October. We estimated whether the differences between groups were significant by calculating the 95% CI as:


$$\begin{array}{l} E_{1}-E_{2}\pm 1.96\sqrt{{\left(SE_{1}\right)}^{2}+{\left(SE_{2}\right)}^{2}} \end{array}$$

where E_1_ and E_2_ are the estimates for the two groups, and SE_1_ and SE_2_ are their corresponding standard errors (29).

### Sensitivity analyses

Single-day lag effect estimates might underestimate the cumulative association between DTR and ARLI; we therefore, used lag05 of DTR for our main analyses. A set of sensitivity analyses were applied to confirm the robustness of our main results. The main results were firstly estimated by altering the degree of freedom for temporal trends and meteorological variables (degrees of freedom alternating from five to eight) (30, 31). Second, air pollutants were further adjusted in models. Last, we have transformed the meteorological data into a standard normal distribution by subtracting the mean and dividing by the standard deviation.

The results in this study were reported as excess relative risk (ER) and 95% CI. All the statistical analyses were conducted in R version 4.1.0 (32). A *p*-value < 0.05 was considered the statistically significant.

## Results

### Characteristics of the study sample

Table 1 presents the mean, standard deviations (SDs), and percentiles of daily ALRI outpatient visits, meteorological variables, and concentrations of air pollutants in our study sample. There was a total of 79,416 hospitalizations with ALRI during the study period, in which 27,427 (33.4%) were due to pneumonia and 50,401 (64.6%) were due to bronchiolitis. There were on average 38 ALRI hospitalizations (13 pneumonia and 25 bronchiolitis) per day during the study period. The mean DTR during the study period was 9 (SD: 3.4) °C, with the median DTR of 8.6 (interquartile range: 6.5 to 11.5) °C. DTR was negatively correlated with daily temperature and relative humidity, while it was positively correlated with daily concentrations of air pollution (PM_2.5_, PM_10_, NO_2_, and SO_2_). The matrix of pairwise Pearson correlation coefficients between the environmental variables are shown in Supplementary Table S1.

**Table 1:** Summary statistics of acute lower respiratory infections outpatient visits, meteorological variables, and air pollutants.

	**Mean**	**SD**	**Percentile**				
			**Min**	**25^th^**	**50^th^**	**75^th^**	**Max**
**No. of daily outpatient visits**							
ALRI	38	18	1	25	35	47	124
Pneumonia	13	10	0	7	11	17	73
Bronchiolitis	25	11	0	16	23	31	70
**Meteorological variables**							
Temperature, °C	22.3	5.8	1.8	18.2	24.0	27.2	30.7
DTR, °C	9.0	3.4	2.1	6.5	8.6	11.5	20.2
Relative humidity, %	80.3	11.2	34.0	74.4	82.7	88.9	97.0
**Air pollution**							
PM_2.5_, μg/m^3^	35.3	19.1	4.6	21.4	30.8	45.1	154.5
PM_10_, μg/m^3^	56.6	27.2	10.0	37.2	50.0	71.2	216.2
SO_2_, μg/m^3^	11.0	4.8	2.6	7.6	10.1	13.5	37.7
NO_2_, μg/m^3^	46.0	18.5	8.8	33.3	41.9	54.3	176.7
O_3_, μg/m^3^	49.6	27.8	3.5	27.9	45.9	66.4	189.0

### DTR is associated with an increased risk of ALRI outpatient visits

**Figure 1:**
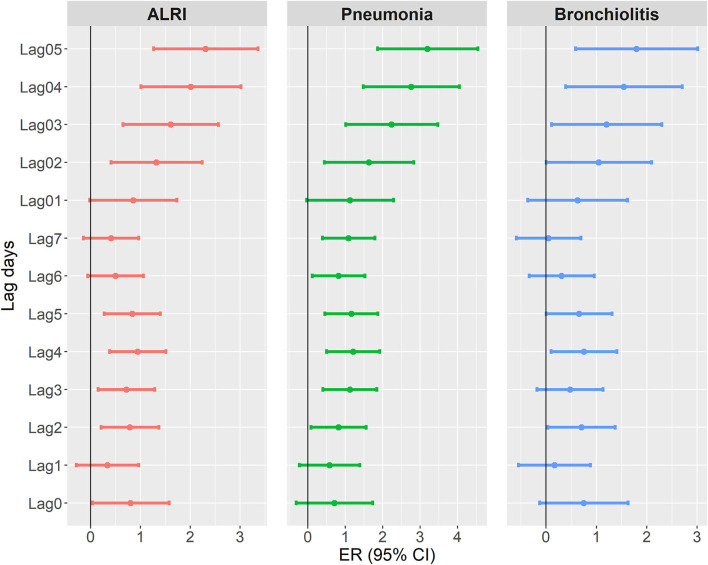
Excess risk (and 95% confidence interval) of acute lower respiratory outpatient visits per 1°C increment in diurnal temperature range at different lag days (the number of days between exposure and the estimated effect).

Figure 1 shows the ER and 95% CI of outpatient visits of ALRI, pneumonia, and bronchiolitis per one unit increment in DTR at various lag days (lag 0 to lag 7 and moving averages of lag 01 to lag 05). The results show that moving average of DTR at 5 days (lag 05) was significantly associated with an elevated risk of ALRI outpatient visits: the ER (95% CI) estimates were: 2.31 (1.26, 3.36) for ALRI, 3.19 (1.86, 4.54) for pneumonia, and 1.79 (0.59, 3.01) for bronchiolitis. The significant patterns were generally consistent at higher lag days (lag 2 to lag 5 and lag 02 to lag 05), while the results were insignificant at lag 0 to lag 1 and lag 01 to lag 02.

**Figure 2:**
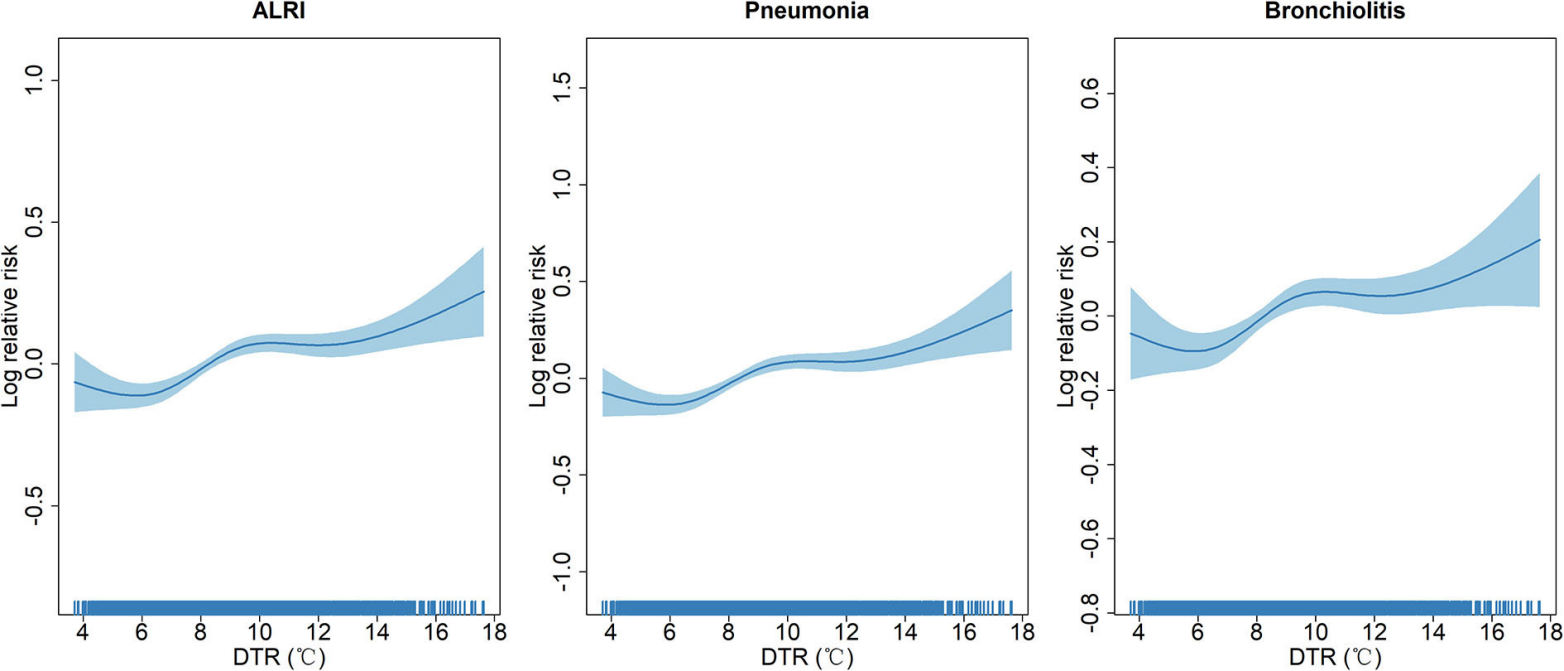
Concentration-response curves showing the non-linear association between diurnal temperature range and log relative risks of acute lower respiratory infection outpatient visits.

To further understand potential non-linear relationships between DTR and risk of ALRI (pneumonia and bronchiolitis) outpatient visits, we constructed non-linear spline models to estimate the concentration-response curves for this relationship. Figure 2 reveals similarly close-to-linear relationships between DTR and log relative risks of ALRI, pneumonia, and bronchiolitis in the sample, although there were slight inverse-U relationships in the DTR range of 8 to 12°C.

### Sensitivity analyses

We conducted a set of sensitivity analyses to check the robustness of the main results. First, we tested the influence of the dfs for temporal trends and meteorological variables on the main results by alternating them from five to eight. The results (Supplementary Table S2) suggest that the changing dfs for temporal trends or meteorological variables did not nullify the significance o or change the direction of the ER estimates. Second, we tested the association between DTR and ALRI outpatient visits by further adjusting for concentrations of daily air pollutants. Supplementary Table S3 indicates that the pattern of associations was consistent with the main results. At last, we reconstructed our models by adjusting normalized meteorological variables to tackle potential issues caused by extreme values and the results agreed with the main findings (Supplementary Figure S1).

### Associations between DTR and ALRI outpatient visits by subgroups

We further examined the associations between DTR and ALRI outpatient visits by sex, age group (<5 years and 5 to 14 years), and seasons to test for potential effect modification. Table 2 shows significant effect modification by seasons (rainy and dry seasons), while the ER and 95% CI estimates were not significantly different across sex and age groups. The associations between DTR and outpatient visits of ALRI as well as pneumonia and bronchiolitis were significantly larger during rainy seasons (ER for ALRI: 3.02%, 95% CI: 1.43, 4.64%) than those in dry seasons (ER for ALRI: 2.21%, 95% CI: 0.65, 3.81%).

**Table 2:** Excess risk and 95% confidence intervals of ALRI, pneumonia, and bronchiolitis for each 1°C increments in DTR stratified by gender, age group, and season.

**Stratum**	**ER (95% confidence interval), %**		
	**ALRI**	**Pneumonia**	**Bronchiolitis**
**Sex**			
Male	2.29 (1.14, 3.46)	3.98 (2.36, 5.62)	1.75 (0.40, 3.11)
Female	2.31 (1.03, 3.61)	2.14 (0.44, 3.88)	2.34 (0.80, 3.90)
**Age**			
<5	2.22 (1.15, 3.31)	2.99 (1.62, 4.37)	1.87 (0.61, 3.14)
5–14	2.53 (0.67, 4.43)	3.92 (0.72, 7.22)	2.13 (0.13, 4.18)
**Season**			
Rainy	**3.02 (1.43, 4.64)**	**3.46 (1.44, 5.52)**	**2.75 (0.79, 4.75)**
Dry	**2.21 (0.65, 3.81)**	**2.75 (0.82, 4.72)**	**1.93 (0.20, 3.69)**

The bold texts represent the statistically significant differences (*p* < 0.05).

## Discussion

In this large time-series study of 79,416 ALRI outpatient visits spanning seven years in Guangzhou, China, we found that higher DTR was associated with significantly higher risks of outpatient visits of ALRI, pneumonia, and bronchiolitis. The results were consistent when alternative dfs were applied to temperature and relative humidity, as well as daily concentrations of air pollution were adjusted for. Further spline analyses suggested close-to-linear relationships between DTR and ALRI outpatient visits. We also found significant effect modification by season, where the ERs were significantly larger during rainy seasons than those in dry seasons.

Numerous studies have investigated the associations of temperature and its variability with the incidence of respiratory diseases (33–36). For example, Lim, Hong, and Kim estimated that each 1°C increment in DTR was associated with a 1.1% (95% CI: 0.1 to 2%) increase in asthma hospital admissions in four metropolitan areas in Korea (34). However, these previous studies primarily focused on the occurrence of respiratory disease among adults, and little to no studies investigated the outpatient visits of ALRI among children under the age of 5 years. Our study closes the unfilled knowledge gap between DTR and ALRI outpatient visits among children and reconfirms that positive associations exist in accordance with previous studies. Our estimates of ERs were generally larger than those in previous studies, which may be attributable to the more vulnerable population (children under the age of 14) in this study.

Although this time-series epidemiological study does not illustrate the physiological mechanisms underlying associations between DTR and ALRI, several biological mechanisms could underpin our observed relationships. Dramatic temperature change can afflict the circulation, immune, and thermoregulatory system, causing abnormality of several laboratory indicators including heart rate, blood pressure, blood viscosity, and oxygen levels (15, 37–39). Changes in these physiological measures may further lead to incidence and outpatient visits of ALRI among children. These mechanisms also increase the possibility of interactive seasonal and weather effects as they influence the health outcomes *via* similar pathways (40).

The effect modification of the associations between DTR and respiratory diseases by season is also reported by several previous studies (20, 21, 41, 42). For example, a time-series study of 445 communities in 20 countries and regions has reported that temperature amplifies all-cause mortality attributable to DTR (15). Warm seasons coupled with elevated levels of DTR, could cause circulatory and immune system disorders (37) and increase the vulnerability of people with immature immune system. Our findings support this effect modification in a vulnerable population of children under the age of 14, and this has not been reported by previous studies. However, we also noticed that a few other studies reported that cold seasons, instead of warm seasons, enhances this association (35, 43). This inconsistency in literature may be caused by the underlying population, exposure measurement, or regional differences, and could be a direction for future research.

Although DTR in China showed a slightly decreasing trend in the recent few decades (44, 45), the severe heat waves over these years make the temporal trend of DTR less certain. The effect of DTR on outpatient visits of ALRI in children in the context of extreme heat waves is an important topic and direction for future research (46). Evidence-based health policies are required to be formulated to mitigate the adverse health effects of DTR in a rapidly changing and exacerbating climate.

A few limitations should be noted for this study. First, this is a time-series analysis of daily counts of outpatient visits and the results are subject to potential ecological fallacy bias (47). Second, this is a single-city study conducted in Southern China, so the results may not be generalizable to other cities in China, particularly those in Northern China with different patterns of DTR and seasonality. Third, a few covariates including socioeconomic status and distance to hospitals were not available to the study team, which may lead to confounder bias. Fourth, since we were not able to access data on individual addresses, there may be a small proportion of patients who lived in other cities but sought care in our sample hospital.

Our study included complete data on ALRI outpatient visits spanning a consecutive 8 years from a large tertiary hospital, and the fair amount of sample yielded large statistical power and generated significant model results. The public health implications of this study are that the parents or guardians of children need to be aware of temperature change and adopt effective measures to minimize the adverse health effects of DTR on the occurrence of respiratory diseases among children.

## Conclusion

In summary, this multi-year time-series study suggests that DTR may increase the outpatient visits of ALRI, pneumonia, and bronchiolitis among children. The effects of DTR may be stronger during rainy seasons. Relevant health policies, including health education and promotion, are needed to mitigate the deleterious health impacts.

## Data availability statement

The data analyzed in this study is subject to the following licenses/restrictions: We do not have permission to share data. Requests to access these datasets should be directed to XZ: zhangxiaogd2h@163.com.

## Author contributions

ZL and XZ: investigation and visualization. QM, ZL, and XZ: supervision. ZZ: writing—original draft. ZZ, DX, JC, and QM: writing—review and editing. All authors contributed to the article and approved the submitted version.

## Conflict of interest

The authors declare that the research was conducted in the absence of any commercial or financial relationships that could be construed as a potential conflict of interest.

## Publisher's note

All claims expressed in this article are solely those of the authors and do not necessarily represent those of their affiliated organizations, or those of the publisher, the editors and the reviewers. Any product that may be evaluated in this article, or claim that may be made by its manufacturer, is not guaranteed or endorsed by the publisher.
