# Supplementary material for: Daily diurnal temperature range associated with outpatient visits of acute lower respiratory infection in children: A time-series study in Guangzhou, China
Source: Front Public Health. 2022 Oct 20;10:951590. doi: 10.3389/fpubh.2022.951590 (PMC9632279; doi:10.3389/fpubh.2022.951590)
Supplement: Supplementary file 1 [file Data_Sheet_1.pdf]

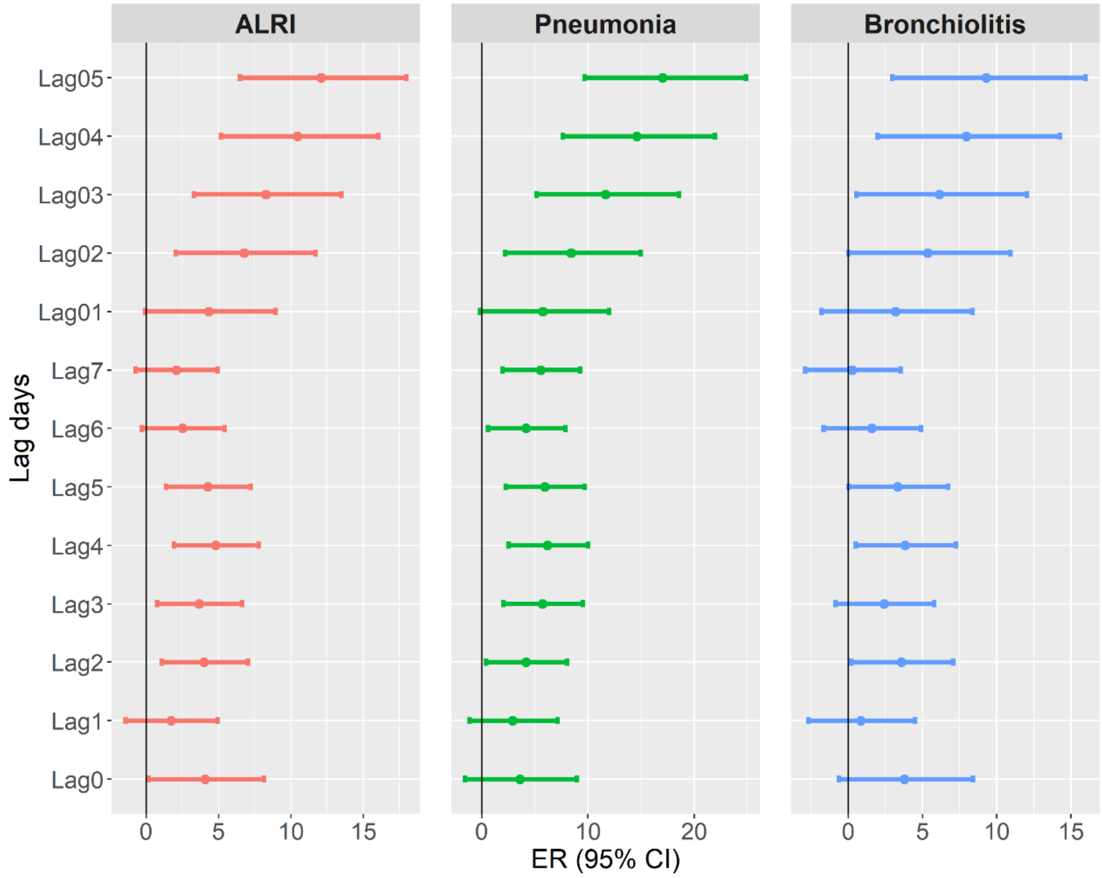

Figure S1. Excess risk (and 95% confidence interval) of acute lower respiratory outpatient visits per one SD (3.41 ℃) increment in diurnal temperature range at different lag days (the number of days between exposure and the estimated effect).

Table S1. Pearson correlation coefficients between the environmental factors.

	DTR	Temperature	RH	$PM_{2.5}$	PM_{10}	NO_2	SO_2
DTR	1.00	-	-	-	-	-	-
Temperature	**-0.54**	1.00	-	-	-	-	-
RH	**-0.82**	**0.61**	1.00	-	-	-	-
$PM_{2.5}$	**0.48**	**-0.23**	**-0.42**	1.00	-	-	-
PM_{10}	**0.53**	**-0.19**	**-0.46**	**0.96**	1.00	-	-
NO_2	**0.47**	**-0.21**	**-0.33**	**0.75**	**0.79**	1.00	-
SO_2	**0.30**	-0.04	**-0.31**	**0.67**	**0.67**	**0.50**	1.00

Bold present statistically significant ($P<0.05$).

Table S2. Associations between DTR and risks of ALRI outpatient visits using different degrees of freedom for spline effects of temporal trends and temperature.

Models	ALRI	Pneumonia	Bronchiolitis
df=5 for temporal trends	2.10 (0.95, 3.25)	2.72 (1.28, 4.19)	1.86 (0.61, 3.13)
df=7 for temporal trends	1.64 (0.56, 2.72)	2.42 (1.05, 3.81)	1.42 (0.16, 2.69)
df=8 for temporal trends	1.69 (0.64, 2.75)	2.36 (1.01, 3.73)	1.63 (0.38, 2.89)
df=4 for temperature	1.74 (0.69, 2.80)	2.45 (1.10, 3.82)	1.60 (0.36, 2.86)
df=5 for temperature	1.74 (0.69, 2.80)	2.46 (1.11, 3.83)	1.63 (0.38, 2.90)
df=6 for temperature	1.75 (0.70, 2.81)	2.39 (1.03, 3.78)	1.68 (0.43, 2.95)

Table S3. Associations between DTR and risks of ALRI outpatient visits after adjusting for concentrations of air pollution.

Models	ALRI	Pneumonia	Bronchiolitis
Adjusting for $PM_{2.5}$	1.35 (0.26, 2.47)	1.69 (0.33, 3.07)	1.29 (0.01, 2.60)
Adjusting for PM_{10}	1.22 (0.12, 2.33)	1.54 (0.19, 2.92)	1.14 (-0.15, 2.45)
Adjusting for NO_2	1.23 (0.13, 2.34)	1.59 (0.23, 2.97)	1.10 (-0.19, 2.40)
Adjusting for SO_2	1.33 (0.25, 2.42)	1.91 (0.55, 3.28)	1.27 (-0.01, 2.57)
